# Identifying optimal reference genes for the normalization of microRNA expression in cucumber under viral stress

**DOI:** 10.1371/journal.pone.0194436

**Published:** 2018-03-15

**Authors:** Chaoqiong Liang, Jianjun Hao, Yan Meng, Laixin Luo, Jianqiang Li

**Affiliations:** 1 Department of Plant Pathology, China Agricultural University/Key Laboratory of Plant Pathology, Ministry of Agriculture, Beijing, China; 2 Department of Plant and Microbial Biology, University of California, Berkeley, Berkeley, California, United States of America; 3 Plant Gene Expression Center, United States Department of Agriculture, Agricultural Research Service, Albany, California, United States of America; 4 School of Food and Agriculture, The University of Maine, Orono, Maine, United States of America; 5 Beijing Key Laboratory of Seed Disease Testing and Control, China Agricultural University, Beijing, China; Chinese Academy of Agricultural Sciences, CHINA

## Abstract

*Cucumber green mottle mosaic virus* (CGMMV) is an economically important pathogen and causes significant reduction of both yield and quality of cucumber (*Cucumis sativus*). Currently, there were no satisfied strategies for controlling the disease. A better understanding of microRNA (miRNA) expression related to the regulation of plant-virus interactions and virus resistance would be of great assistance when developing control strategies for CGMMV. However, accurate expression analysis is highly dependent on robust and reliable reference gene used as an internal control for normalization of miRNA expression. Most commonly used reference genes involved in CGMMV-infected cucumber are not universally expressed depending on tissue types and stages of plant development. It is therefore crucial to identify suitable reference genes in investigating the role of miRNA expression. In this study, seven reference genes, including *Actin*, *Tubulin*, *EF-1α*, *18S rRNA*, *Ubiquitin*, *GAPDH* and *Cyclophilin*, were evaluated for the most accurate results in analyses using reverse transcription-quantitative polymerase chain reaction (RT-qPCR). Gene expression was assayed on cucumber leaves, stems and roots that were collected at different days post inoculation with CGMMV. The expression data were analyzed using algorithms including delta-Ct, geNorm, NormFinder, and BestKeeper as well as the comparative tool RefFinder. The reference genes were subsequently validated using miR159. The results showed that *EF-1α* and *GAPDH* were the most reliable reference genes for normalizing miRNA expression in leaf, root and stem samples, while *Ubiquitin* and *EF-1α* were the most suitable combination overall.

## Introduction

Cucumber (*Cucumis sativus*) is an economically important member of the gourd family, Cucurbitaceae, that is cultivated throughout the world [1, 2]. Cucumber yield is significantly affected by a range of biotic and abiotic stresses, and viral diseases constitute a major constraint to both cucumber quality and yield [3]. *Cucumber green mottle mosaic virus* (CGMMV) is one of the important viral pathogens of cucumber. CGMMV seriously affects the host’s physiology, leading to sterile flowers and abnormal fruit [4], which often results in reduced yields and a lowered market value [5]. As a consequence, strict quarantine regulations have been imposed to prevent the spread of CGMMV between different regions of cultivation, and limit its negative effect on global cucumber production [6, 7].

It is now well known that microRNAs (miRNAs), a class of small, non-coding, single stranded RNA molecules that range in the length from 19 to 24 nucleotides [8], play a fundamental role in key biological processes in plants including responses to environmental stresses and host-pathogen interactions [9–11]. Recent research has demonstrated that miRNA expression can be used as an indicator of cucumber responses to viral infection, which provides a new insight into the complex regulatory networks and specific miRNA involved in the metabolic and signaling pathways underlying developmental and cellular processes in cucumber infected by viroid [12] or virus [13]. For example, Liu et al. [13] identified 8 novel, 23 known and 127 putative candidate miRNAs with altered expression in CGMMV-infected cucumber. However, accurate quantification of miRNA expression levels in response to viral infections requires reliable internal controls with highly stable expression. Using reference genes that can be used universally in a wide range of tissues can greatly improve the efficacy of this kind of analysis. Indeed, the selection of suitable reference genes is a pre-requisite for any expression study, in order to minimize experimental errors associated with different conditions and samples [14–17]. Constitutively expressed housekeeping genes are transcribed at a relatively constant level, and are usually selected for this purpose [18, 19], because their expression is generally assumed to be minimally affected by experimental conditions. However, recent studies have shown that housekeeping genes that are commonly used as reference genes may not be as consistently expressed as previously thought [20–23], and can vary according to plant species, tissue type, and environmental conditions [24]. The use of non-validated references genes can therefore result in inaccurate quantification of miRNA expression when comparing samples from different sources.

Reverse transcription-quantitative polymerase chain reaction (RT-qPCR) has become an extremely powerful tool for the detection and quantification of miRNA expression, exhibiting a high degree of sensitivity and specificity [25, 26]. Although suitable reference genes are well established in wheat [27], citrus [28], castor bean [29] and honeysuckle [30], there have been only three studies validating candidate reference genes for normalizing expression in cucumber. Furthermore, although these studies investigated a range of test conditions including abiotic stresses (nitrogen, salinity, drought, osmotic and oxidative stress, heat or cold), biotic stress (*Pseudoperonospora cubensis*) and hormone treatment (salicylic acid, methyl jasmonic acid and abscisic acid) [31–33], there have been no similar evaluations of suitable reference genes for the expression of miRNA in cucumber infected by viruses.

Given the increasing evidence that the expression of genes commonly used as internal controls for RT-qPCR studies can respond differently under different treatment conditions [34], the systematic evaluation of reference genes derived from genome-wide analyses is becoming an essential component of RT-qPCR analyses in order to improve the reliability of the results [19, 35, 36]. Many statistical algorithms have been developed to evaluate the expression stability of reference genes. For example, geNorm [34], NormFinder [37] and BestKeeper [38], which are all Visual Basic Applets for Microsoft Excel, have been used to determine the expression stability of candidate reference genes in a wide variety of plants including *Arabidopsis* [19, 39, 40], potato [41, 15], rice [42,43], citrus [28], soybean [44]and cucumber [31–33]. However, differences between the results generated by these algorithms have sometimes been observed [45, 46], indicating that a combination of several algorithms could provide a more accurate analysis.

Previous studies have identified a variety of reference genes for use in a range of cucumber tissues and organs at different developmental stages and under different biotic and abiotic stresses [31–33]. The most widely used internal controls include the genes encoding actin and tubulin, which are cytoskeletal proteins [18]; elongation factor 1-alpha (EF-1α), which facilitates translational elongation [47]; 18S ribosomal RNA (18S rRNA), a part of the ribosomal functional core [18]; ubiquitins, which are involved in the degradation of cellular proteins [48]; glyceraldehyde 3-phosphate dehydrogenase (GAPDH), which is involved in glycolysis [18]; and cyclophilin, a specific cytosolic binding protein for cyclosporin A [49]. However, there is currently little data regarding the expression stability of these reference genes in cucumber infected with viruses such as CGMMV. The object of the current study was therefore to investigate seven commonly used references genes including *Actin*, *Tubulin*, *EF-1α*, *18S rRNA*, *Ubiquitin*, *GAPDH* and *Cyclophilin* to discover which are the most reliable internal controls for miRNA expression studies in a range of cucumber tissues infected with CGMMV.

## Materials and methods

### Plant materials and virus inoculant

CGMMV-free cucumber seeds cv. ‘Zhongnong 16’) [50] were obtained from the Institute of Vegetables and Flowers at the Chinese Academy of Agricultural Sciences (Beijing, China). The seed was sown in an insect-proof greenhouse in accordance with the protocol of a previous study [4]. CGMMV was originally collected from Zhejiang province, China, and was propagated and maintained *in vivo* on a cucumber host (Beijing Key Laboratory of Seed Disease Testing and Control, Beijing, China). Cucumber seedlings were artificially inoculated with either CGMMV sap (Treatment samples) or a Phosphate buffer/tween 80 mixture (PBST) (Control) at the three-true-leaf stage. Samples from the leaves, stems and roots of the test plants that were collected at 1, 14, 28 and 42 days post-inoculation (dpi) respectively, immediately frozen in liquid nitrogen and stored at -80°C until use.

### Total RNA extraction and cDNA synthesis

Total RNAs were extracted from the cucumber samples using TRIzol^®^ Reagent (Ambion, USA) according to the protocol of the manufacturer, and its quantity and quality assessed using a NanoDrop 2000 (Thermo Scientific). RNA samples exhibiting an A_260/A280_ ratio of 1.8–2.0 and an A_260/A230_ ratio of 2.0–2.2 were selected for the subsequent analysis. cDNA was synthesized using the miRcute miRNA First-Strand cDNA Synthesis Kit (KR201, Tiangen Biotech, China) and stored at -20°C until use (http://dx.doi.org/10.17504/protocols.io.naxdafn).

### Amplification of gene transcripts

The expression stability of seven reference genes, including *Actin*, *Tubulin*, *EF-1α*, *18S rRNA*, *Ubiquitin*, *GAPDH* and *Cyclophilin* was evaluated in cucumber leaf, stem and root samples collected at different time points post inoculation with CGMMV. According to sequences of seven reference genes (S1 File), specific primers for RT-qPCR were designed using the Primer Premier 5.0 software, with the product size restricted to 127 to 193 base pairs (bp) and an optimal melting temperature (Tm) of 60°C (Table 1). The miR159 forward primer was designed using a csa-miR159 specific sequence, while the universal primer from the miRcute miRNA qPCR Detection Kit (SYBR Green) (FP401, Tiangen Biotech, China) was used as the reverse primer.

**Table 1:** Primers used to amplify partial sequences of 7 reference genes and miR159 from cucumber samples.

GenBank accession number	Gene	Annotation	Sequence of forward/reverse primers (5’-3’)	Amplicon length (bp)	Tm^a^ (°C)	PCR efficiency (E)	Correlation coefficient (R^2^)
AB010922	*Actin*	Actin	ATGACGCAGATAATGTTTGAG/ GGAGAATGGCATGAGGGAGGG	175	60	96.69	0.9976
AJ715498	*Tubulin*	Alpha-tubulin	CAAGGAAGATGCTGCCAATAA/CCAAAAGGAGGGAGCCGAGAC	179	60	101.91	0.9970
EF446145	*EF-1α*	Elongation factor 1-alpha	ACTGGTGGTTTTGAGGCTGGT/CTTGGAGTATTTGGGTGTGGT	135	60	100.70	0.9992
AF206894	*18S rRNA*	18S ribosomal RNA	CAAAGCAAGCCTACGCTCTGT/CTATGAAATACGAATGCCCCC	127	60	91.45	0.9973
AF104391	*Ubiquitin*	Ubiquitin-like protein (UBI-1)	CTAATGGGGAGTGGGGAAGTA/GTCTGGATGGACAATGTTGAT	135	60	90.17	0.9991
NM001305758	*GAPDH*	Glyceraldehyde-3-phosphate dehydrogenase	GCCTTGGTCCTCCCTTCTCTT/ATGCAGCATTCACCTCTTCAG	133	60	96.03	0.9996
AY942800	*Cyclophilin*	Cyclophilin (M2)	GCTGGACCTGGAACCAACGGA/ TCTAAGAGAGCTGGCCACAAT	193	60	90.44	0.9976
-	miR159	csa-miR159	TTTGGATTGAAGGGAGCTCTA	80	60	93.50	0.9991

RT-qPCR was conducted using the miRcute miRNA qPCR Detection Kit (SYBR Green) (FP401, Tiangen Biotech, China) and 20 μL reaction mixtures containing 1 μL template cDNA (corresponding to 600 ng of starting total RNA), 10 μL 2× miRcute miRNA Premix (with SYBR & ROX), 0.4 μL of each primer (10 μM) and 8.2 μL RNase-free water. The RT-qPCR was processed using the Applied Biosystems 7500 Fast Real-Time PCR System (Life Technologies, USA) with the following program: 94°C for 2 min, followed by 40 cycles of 94°C for 20 s, 60°C for 34 s. Melting curves were generated at 95°C after the reaction had been terminated (S1 Fig). A set of seven 4-fold cDNA dilutions were used to create standard curves, and the correlation coefficient (*R*^*2*^) and PCR efficiency (*E*) determined using a linear regression model: *E* = (10^−1/slope^ −1)×100 [51] (S2 Fig). Each RT-qPCR reaction was performed in triplicate (3 technical replicates) for each sample (3 plants per sample). Non-template controls produced undetectable cycle threshold (Ct) values, while subsequent electrophoresis on 2.0% agarose gel stained with ethidium bromide confirmed the size of RT-qPCR products amplified from the treatment samples (S3 Fig).

### Expression stability of reference genes

Expression stability of each reference gene was evaluated using four subsets of the samples, including leaf, stem, and root samples as well as a composite sample that incorporated the data from all of the individual samples to provide an overall assessment. The stability of the seven reference genes was initially evaluated individually using four different algorithms, including comparative delta-Ct (ΔCt) [52], geNorm version 3.5 [34], NormFinder version 20 [37] and BestKeeper version 1 [38], before the comparative web-based tool, RefFinder (http://leonxie.esy.es/RefFinder/) was used to collate the results from all four algorithms.

The comparative delta-Ct algorithm compares and ranks candidate reference genes by comparing the relative expression of ‘pairs of genes’ within each sample. If the ΔCt value of the two genes remains constant among different samples, it means that either both genes are stably expressed in those samples, or that the genes are co-regulated. However, if the ΔCt fluctuates, then one or both genes are variably expressed. The introduction of a third, fourth, or fifth gene into the comparisons provides increased fidelity indicating which pairs show the least variability, and hence which gene(s) has the most stable expression among test samples. These results can then be ranked or discarded. This process of elimination strategy allows a relatively large number of genes to be compared against one another according to their ΔCt values [52].

The geNorm algorithm also compares pairs of reference genes calculating expression stability measure *M*, which represents the average pairwise variation of each gene with the other candidate genes, and allows the selection of two optimized reference genes through the sequential exclusion of genes with lower expression stability [34]. The lower the *M* value, the higher the expression stability of a particular gene. In addition, geNorm provides analytical data regarding the optimal number of reference genes required to provide reliable normalization (V_n/n+1_). According to Vandesompele et al. [34], a variation of < 0.15 indicates that an additional reference gene provides no significant improvement to the normalization factor, which means that the optimal number of reference genes has already been achieved and that the inclusion of the additional reference gene is not required.

In contrast, the NormFinder algorithm calculates and ranks the expression stability of each reference gene according to comparisons made between intra-group and inter-group variations of the candidate genes under evaluation [37].

Based on the output from geNorm and NormFinder, the two poorest performing reference genes were eliminated, and the rest five genes were investigated further for expression stability using BestKeeper [38], which estimates the most appropriate reference genes by computing geometric mean of a candidate cDNA based on its crossing point (CP) or threshold cycle (Ct) values. In addition, BestKeeper also takes into account the raw data from the RT-qPCR analysis. The algorithm calculates a pairwise correlation coefficient between each gene and the BestKeeper index (BI) and a standard deviation (SD) of the Ct-values for the whole data set. The gene with the highest correlation coefficient is considered to have the most stable expression [33, 38].

The data of expression stability was analyzed further using RefFinder, which can be used to confirm and integrate the output obtained from the comparative delta-Ct, geNorm, NormFinder and BestKeeper algorithms. RefFinder produces a final overall ranking of the reference genes under evaluation based on the geometric mean calculated from the weighting of every gene produced by each of the individual algorithms [53].

### Validation of selected reference genes using miR159 expression

Previous research has shown that miR159 responds to the stress of both *Turnip yellow mosaic virus* (TYMV) and *Turnip mosaic virus* (TuMV) in *Arabidopsis thaliana* [54]. Given that our own research has shown that miR159 could also be important in the cucumber response to CGMMV [13], it was selected as the best candidate miRNA to assess the reference genes evaluated in the bioinformatics analysis. Both the highest ranked reference genes and combinations of the most and least stable genes were evaluated. In addition to miR159, 8 other miRNAs (miR169, miR172, miR838, miR854, miR2673, miR5637, miR5658 and csa-miRn6-3p) were also evaluated to further investigate which combination of reference genes performed best in a variety of circumstances (S4 Fig). Plants were sampled at 1, 14, 28 and 42 dpi. The ratio of relative expression of miR159 in CGMMV-infected samples were compared to unstressed control and differential expression assessed using the combination of different reference genes. The generated data was normalized using the mathematic model described in REST 2009 software (http://REST.gene-quantification.info/) [55]. The ratio of relative expression of target gene was computed, based on its real-time PCR efficiencies (E) and the Ct difference (Δ) of one treatment sample (average Ct value) versus one control (average Ct value) (ΔCt _treatment—control_). For example, the expression ratio of miR159 was estimated using its average Ct value at 1, 14, 28 and 42 dpi in leaf tissues. The calculation formula (Equation 3.4) was as follows:

$$Ratio={\left(E_{miRNA}\right)}^{\Delta Ct\;miRNA\;\left(average\;treatment‑average\;control\right)}/{\left(E_{reference\;gene}\right)}^{\Delta Ct\;reference\;gene\;\left(average\;treatment‑average\;control\right)}$$

## Results

### Performance of primers and expression stability of reference genes in cucumber

Melting curve and agarose gel electrophoresis analysis showed that a specific fragment of the expected size and a single peak were observed, respectively, in reverse transcriptase-PCR (RT-PCR) and RT-qPCR amplifications (S1 and S3 Figs).

**Fig 1:**
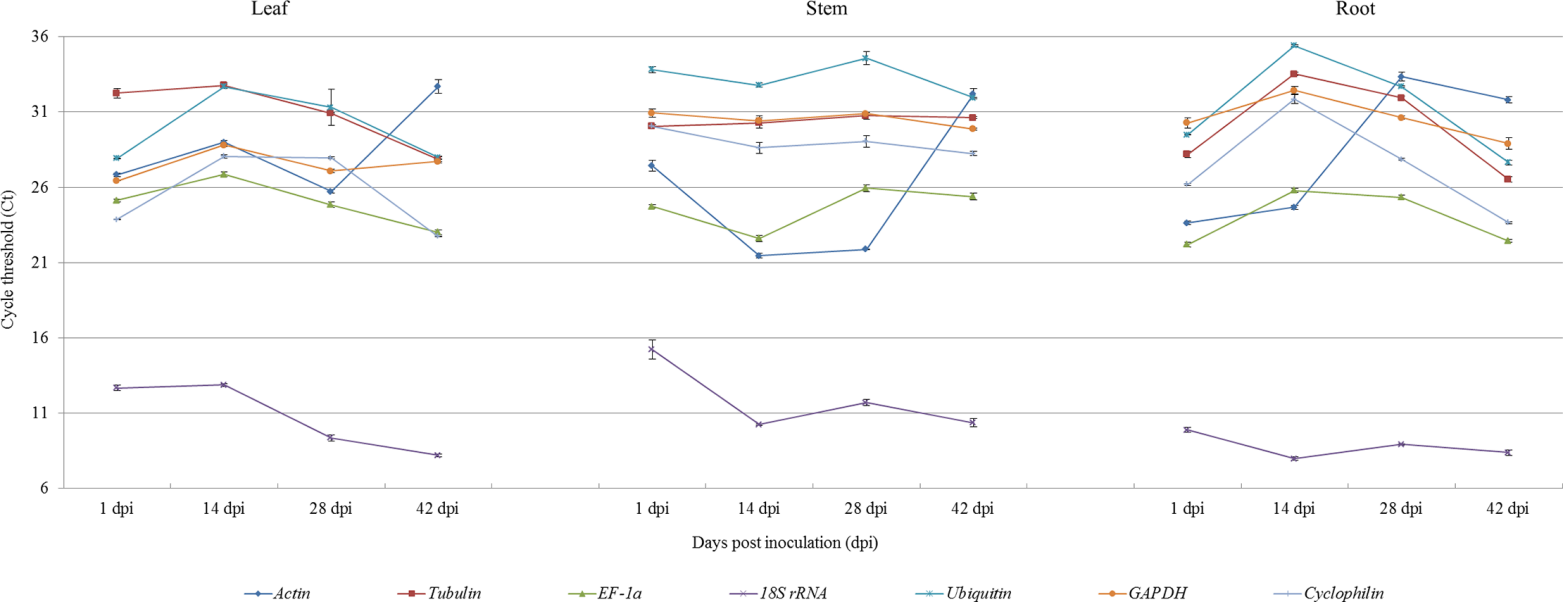
RT-qPCR cycle threshold values of seven reference genes in cucumber leaf, stem and root samples at different time points post-inoculation with CGMMV. Error bars represent the mean of three technical replicates ± SD.

The RT-qPCR analysis revealed that the seven reference genes under evaluation displayed different levels of expression in cucumber (Fig 1). Although expression levels varied according to tissue type and the period of time post inoculation, most Ct values ranged from 21 to 36. Furthermore, the relative level of expression between the genes remained reasonably constant with *18S rRNA*, which exhibited a low Ct value, being the most highly expressed, and *Ubiquitin*, which had a particularly high Ct value, and thus a low level of expression, being the lowest in expression. The most consistent expression levels were observed with *EF-1α* and *GAPDH*, which had similar patterns of expression in all the tissue types and varied by only 5 Ct points across different time points post inoculation.

### Delta-Ct analysis

The delta-Ct analysis also revealed that the expression stability of the different reference genes, which was represented by the standard deviation (SD), varied. For example, *EF-1α* had the lowest SD in leaf and root samples, while *GAPDH* had the lowest SD in stem tissues. Contrary to expectation, *18S rRNA* and *Actin* exhibited the highest SD values in nearly all the samples tested (Table 2). Taken together, these results indicated that *EF-1α* was the best reference gene for leaf and root tissues, while *GAPDH* was the best for stem tissues, and that *18S rRNA* and *Actin* were not a good choice under any circumstances.

**Table 2:** Expression stability of seven reference genes in cucumber analyzed using the delta-Ct algorithm.

Gene	Leaf		Stem		Root		Overall	
	Stability	Ranking	Stability	Ranking	Stability	Ranking	Stability	Ranking
*EF-1α*	1.886	1	2.178	5	2.346	1	2.420	1
*GAPDH*	2.246	2	1.788	1	2.486	2	2.713	5
*Tubulin*	2.270	3	1.965	3	2.497	3	2.619	3
*Ubiquitin*	2.308	4	2.011	4	2.596	4	2.616	2
*18S rRNA*	2.523	5	2.650	6	3.486	6	3.187	6
*Cyclophilin*	2.622	6	1.788	2	2.797	5	2.703	4
*Actin*	4.311	7	5.293	7	6.009	7	5.271	7

### geNorm analysis

**Fig 2:**
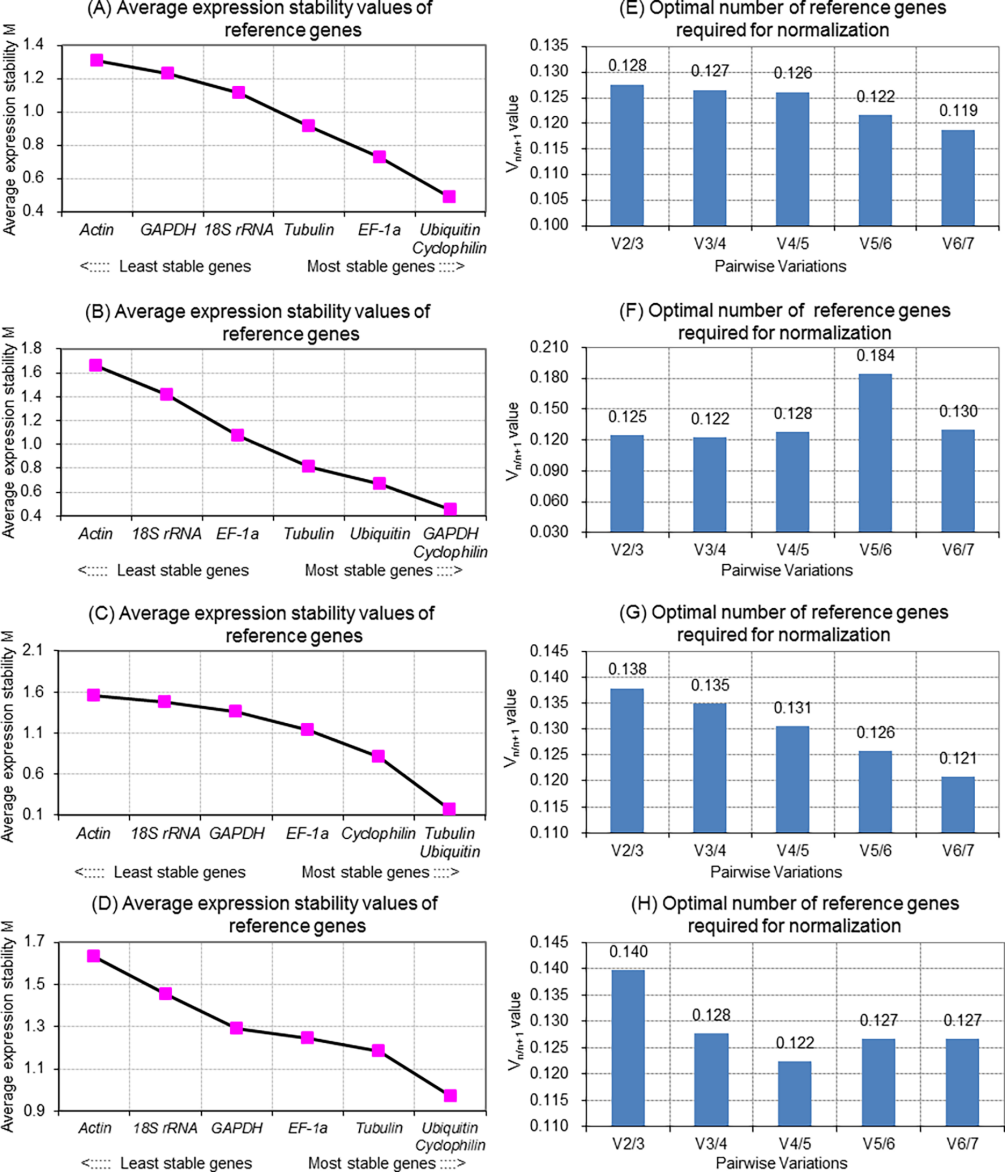
GeNorm data for 5 cucumber reference genes in cucumber leaf, stem and root samples at different time points post-inoculation with CGMMV. (A) to (D) Average expression stability values (M) of cucumber reference genes during stepwise exclusion of the least stable reference gene in different tissue samples. (A) leaf, (B) stem, (C) root, and (D) overall. (E) to (H) Optimal number of reference genes required for accurate normalization of expression based on pairwise variation (V_n/n+1_) analysis of the normalization factors of the reference genes in different tissue samples. (E) leaf, (F) stem, (G) root, and (H) overall.

*M* values of the seven reference genes generated by geNorm produced similar results compared to delta-Ct analysis. In this case, *Ubiquitin*, *Cyclophilin* and *EF-1α* had the highest expression stability in leaves (Fig 2A), *GAPDH*, *Cyclophilin* and *Ubiquitin* in stems (Fig 2B), and *Tubulin*, *Ubiquitin* and *Cyclophilin* in roots (Fig 2C), while *Ubiquitin* and *Cyclophilin* were the most stable overall (Fig 2D). Meanwhile, *Actin* and *18S rRNA* had the lowest expression stability in both stem (Fig 2B) and root tissues (Fig 2C), as well as the lowest stability overall (Fig 2D). Although all the seven references genes exhibited acceptable expression stabilities, the *M* values indicated that in comparison to *Actin*, *Ubiquitin* and *Cyclophilin*, which had relatively low values in all the tissues tested, would be better selections. The geNorm analysis also indicated that if *Ubiquitin* and *Cyclophilin* were used in conjunction, the addition of further reference genes would provide no improvement in fidelity, given that the V_2/3_ value was already below the cut-off (V_n/n+1_<0.15), and therefore that the combination of *Ubiquitin* and *Cyclophilin* would provide reliable normalization of miRNA expression in all CGMMV-infected cucumber samples (Fig 2E, 2F, 2G and 2H).

### NormFinder analysis

The NormFinder results indicated that *EF-1α* was the most stable reference gene in leaf and root tissues, while *Cyclophilin* was the most stable gene in stem tissues (Table 3). In addition, the NormFinder analysis also identified the most suitable reference gene pairings for different tissue samples. Under these circumstances, *Tubulin* and *GAPDH* were the best selection for leaf samples, *EF-1α* and *Cyclophilin* for stem samples, and *EF-1α* and *GAPDH* for root samples, while *EF-1α* and *Ubiquitin* were the best overall (Table 4).

**Table 3:** Expression stability of seven reference genes in cucumber analyzed using the NormFinder algorithm.

Gene	Stability				Intragroup variation			Intergroup variation		
	Leaf	Stem	Root	Overall	Leaf	Stem	Root	Leaf	Stem	Root
*Actin*	0.109	0.131	0.157	0.119	0.025	0.038	0.042	0.054	-0.103	0.049
*Tubulin*	0.035	0.041	0.044	0.042	0.000	0.002	0.002	0.027	-0.031	0.004
*EF-1α*	0.014	0.045	0.022	0.034	0.000	0.001	0.000	0.029	-0.025	-0.004
*18S rRNA*	0.158	0.120	0.100	0.123	0.041	0.028	0.020	0.039	0.099	-0.139
*Ubiquitin*	0.044	0.050	0.045	0.053	0.002	0.002	0.003	-0.038	0.025	0.013
*GAPDH*	0.040	0.043	0.032	0.058	0.002	0.001	0.001	-0.059	0.003	0.056
*Cyclophilin*	0.059	0.033	0.061	0.062	0.006	0.000	0.007	-0.052	0.031	0.021

**Table 4:** Most suitable combination of reference genes for normalizing expression in cucumber according to NormFinder analysis.

Plant tissue	Gene	Stability
Leaf	*Tubulin* + *GAPDH*	0.020
Stem	*EF-1α* + *Cyclophilin*	0.029
Root	*EF-1α* + *GAPDH*	0.019
Overall	*EF-1α* + *Ubiquitin*	0.020

### BestKeeper analysis

The two least suitable genes from the geNorm and NormFinder analyses, *Actin* and *18S rRNA* were excluded from the BestKeeper investigation, which revealed that of the five remaining reference genes, *Ubiquitin* had the highest Pearson coefficient and was therefore the most suitable reference gene regardless of tissue type (Table 5).

**Table 5:** Expression stability of five reference genes in cucumber analyzed using the BestKeeper algorithm.

Tissue	Gene				
	*Tubulin*	*EF-1α*	*Ubiquitin*	*GAPDH*	*Cyclophilin*
Leaf	0.772	0.920	0.946	0.528	0.936
Stem	0.243	0.778	0.841	0.705	0.607
Root	0.992	0.936	0.998	0.957	0.980
Overall	0.774	0.723	0.969	0.691	0.951

### RefFinder analysis

The RefFinder analysis compared the results generated by all of the aforementioned algorithms, and determined that *EF-1α* was the most suitable reference gene for leaf and root samples, *GAPDH* for stem samples, and *Ubiquitin* the most suitable overall (Table 6).

**Table 6:** RefFinder ranking of seven reference genes in cucumber in descending order of expression stability from 1 to 7.

Sample	Rank						
	1	2	3	4	5	6	7
Leaf	*EF-1α*	*Ubiquitin*	*Tubulin*	*Cyclophilin*	*GAPDH*	*18S rRNA*	*Actin*
Stem	*GAPDH*	*Cyclophilin*	*Ubiquitin*	*Tubulin*	*EF-1α*	*18S rRNA*	*Actin*
Root	*EF-1α*	*GAPDH*	*Tubulin*	*Ubiquitin*	*Cyclophilin*	*18S rRNA*	*Actin*
Overall	*Ubiquitin*	*EF-1α*	*Tubulin*	*Cyclophilin*	*GAPDH*	*18S rRNA*	*Actin*

### Validation of selected reference genes by miR159 expression analysis

**Fig 3:**
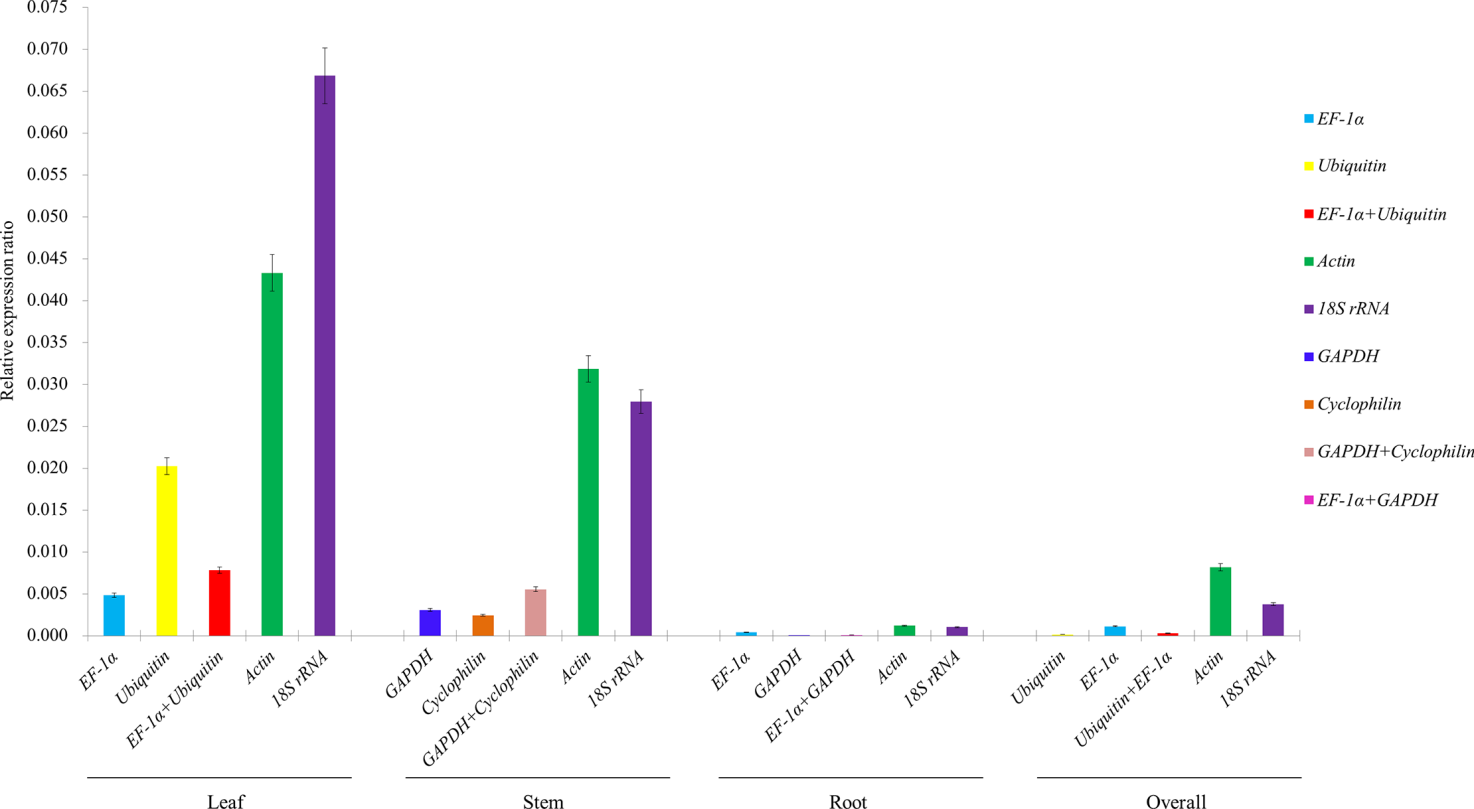
Expression profile of miR159 and validation of selected reference genes in different cucumber tissues infected with CGMMV. Error bars represent the mean of three technical replicates ± SD.

The effectiveness of the reference genes identified as the most stable, most suitable combination and least stable was evaluated in conjunction with miR159, using leaf, stem and root tissues under CGMMV stress. Data normalizations using the most stably expressed reference genes, *EF-1α*, *Ubiquitin* and *GAPDH* resulted in consistent miR159 expression patterns for leaf, stem, root and combined data samples (Fig 3). Similar results were observed when miR159 was compared to stable reference genes in combination, such as *EF-1α* + *Ubiquitin* (0.0078). However, when the least stably expressed reference genes (i.e., *Actin* and *18S rRNA*) were used for data normalization, the expression level of miR159 was considerably biased. For example, higher levels of miR159 expression were observed in leaf samples when *Actin* and *18S rRNA* (0.0433 relative expression ratio for *Actin* and 0.0668 for *18S rRNA*), the least stable reference genes, were used as the internal controls compared to the most stable *EF-1α* (0.0048) and *Ubiquitin* (0.0202). This result indicated that the least stable genes reference *Actin* and *18S rRNA* failed to standardize the expression data effectively. This suggested that the most stably expressed reference genes and most suitable combination showed similar levels of miRNA expression in comparison to the least stable reference genes. Therefore, combinations of stable genes could be utilized for normalization of miRNA under viral stress. Taken together, these results provided a strong evidence reinforcing the observation that the selection of suitable reference genes is critical for obtaining accurate measurements during miRNA expression studies.

## Discussion

We have evaluated and validated the expression stability of seven selected reference genes, including *Actin*, *Tubulin*, *EF-1α*, *18S rRNA*, *Ubiquitin*, *GAPDH* and *Cyclophilin*, to determine the most suitable reference genes used in RT-qPCR analysis of miRNA in leaf, stem and root tissues of cucumber infected by CGMMV. The selection of reliable reference genes is a significant factor influencing the accuracy of comparative expression studies, particularly given that the expression stability of reference genes can vary according to different tissues [24, 31] and experimental conditions such as abiotic and biotic stresses [23]. We have determined that *EF-1α* was the most suitable reference gene for cucumber leaf and root tissues infected by CGMMV, *GAPDH* was the best for stem tissues, and *Ubiquitin* was the best for overall.

Several algorithms have been used for the analysis of expression data relating to the suitability of reference genes, including comparative delta-Ct (ΔCt), geNorm version 3.5, NormFinder version 20 and BestKeeper version 1. We found that these different algorithms selected different genes as the best reference genes, depending on plant tissues evaluated. For example, geNorm predicted that *Ubiquitin* and *Cyclophilin* were the best reference genes for leaves, *GAPDH* and *Cyclophilin* for stems, and *Tubulin* and *Ubiquitin* for roots, while NormFinder indicated that *EF-1α* was the most stable reference gene in leaves and roots, and *Cyclophilin* in stems; BestKeeper resulted in *Ubiquitin* as the most suitable reference gene overall. Such differences are common, and have been observed in many previous studies [31–33, 56–59]. Nonetheless the application of individual algorithms generates extremely useful data that when collated by comparative software such as RefFinder can provide a consensus regarding the expression stability of each reference gene evaluated. The application of this methodology in the current study indicated that *Ubiquitin*and *EF-1α* were the most suitable reference gene combination regardless of tissue types.

Our results were greatly in agreement on *EF-1α* with other studies. For example, *EF-1α* is an extremely common reference gene, and has previously been used to investigate abiotic stresses such as oxidative, heavy metal, salt, osmotic as well as plant growth regulator and biotic stress responses in cucumber [31, 32]. *EF-1α* is also considered as the best reference gene in other plant systems including citrus rootstock under drought stress [60] and castor bean under drought conditions [29], as well as in combination with *Cyclophilin* to study salt stress in potato, and with *18S rRNA* to study late blight [41].

*Actin* and *18S rRNA* are commonly used as reference genes to normalize the quantification of gene expression [20]. For example, they are selected for miRNA expression in sugarcane under cold stress [61], and in wheat infected by *Puccinia graminis* f. sp. *tritici* [62]. However, they were not the most appropriate reference genes in our study, as well as in many other reports. For example, although *Actin* is highly stable in cucumber under cold, heat, drought and salt stress [31], it is the least stably expressed gene in *Arabidopsis* [19], flax [58] and citrus [59]. Furthermore, *Actin* expression can be significantly affected by abiotic and biotic factors [32]. We found that neither *Actin* nor *18S rRNA* exhibited a stable expression pattern, which in turn resulted in much higher values for miR159 expression in cucumber infected with CGMMV compared to *EF-1α*. We agreed with several other studies that *Actin*, *18S rRNA* and *GAPDH* are not the most suitable reference genes [34, 63].

Both *Tubulin* and *GAPDH* have intermediate expression stability compared to the other reference genes in cucumber infected by CGMMV. Several studies have indicated that *Tubulin* is not suitable as a reference gene in cucumber exposed to heavy metals, oxidative, salt and osmotic stress [32] or at extremes of temperature [31], while similar observations have also been made in drought stressed citrus rootstock [60] and citrus exposed to biotic stress [59]. However, some studies have found that under certain circumstances, *Tubulin* can be used as a reference gene, for example, *alpha tubulin* is the most stable reference gene in soybean root tips in responding to Aluminum stress and in soybean roots exposed to heat stress [44], while *beta tubulin* was the most stable reference gene in wheat infected by *Puccinia graminis* f. sp. *tritici* [62].

We found that *Cyclophilin* was one of the most stably expressed reference genes in CGMMV-infected cucumber tissues, which was supported by the study of wheat flag leaves [64], but contraversal to other studies in cucumbers under abiotic or nitrogen stresses, as well as phytohormones [31–33]. We also found that *Ubiquitin* was a robust reference gene, and the most suitable gene for normalizing miRNA expression in cucumber infected by GGMMV. This is in agreement with Warzybok and Migocka, who examined the root and stem of cucumber in response to nitrogen impact [33].

The normalization of expression data can be improved by using combinations of reference genes instead of single genes [34, 65]. We identified three different sets of reference genes optimized to different tissue types, depending on plant tissues. Particularly, *EF-1α* and *Ubiquitin* were the best reference genes in leaf tissues, *GAPDH* and *Cyclophilin* in stems, and *EF-1α* and *GAPDH* in roots, while *Ubiquitin* and *EF-1α* were the best combination overall. The variation observed in these studies confirmed that the selection of references genes optimized for specific biological conditions is an important prerequisite for accurate and reliable normalization of RT-qPCR data. Indeed, the lack of specific reference genes or the use of inappropriate reference genes can result in large variations that skew the interpretation of expression studies.

miR159 belongs to a conserved miRNA family, and plays an important role in cucumber-CGMMV interactions and disease resistance [13]. Given that miR159 has also been implicated in other plant-virus interactions [54] and has great potential as a transgene that confers virus resistance, it was selected as the most suitable target gene to validate the reference genes evaluated in the current study.

The reference genes identified so far appear to be a significant breakthrough in accurately normalizing miRNA expression in virus-infected cucumber samples, and will be of great value when investigating the role of miRNA in the disease resistance and plant-virus interactions of cucumber.

## Conclusions

The selection of reference genes for gene expression study is highly affected by plant tissues, plant toxanomy, and environmental factors and stresses such as pathogen and nitrogen stress. In the cucumber- CGMMV pathosystem, we have determined several individual and combinations of reference genes for the reliable normalization of miRNA expression. *EF-1α* was the most appropriate reference gene for leaf and root tissues, while *GAPDH* was the most suitable reference gene for stems. *Ubiquitin* and *EF-1α* was the most suitable combined references when comparing miRNA expression under a broad range of conditions. Our results could provide a useful foundation reference genes for miRNA expression studies in CGMMV-infected cucumber.

## Supporting information

S1 Fig Melting curves of seven candidate reference genes. (A) to (G) Melting curves of *Actin*, *Tubulin*, *EF-1α*, *18S rRNA*, *Ubiquitin*, *GAPDH* and *Cyclophilin*.
(TIF)

S2 Fig Standard curves of seven candidate reference genes. (TIF)

S3 Fig Electrophoresis of PCR products corresponding to seven candidate reference genes with expected sizes on an agarose gel. (A) Amplification of reference genes with gradient annealing temperatures (from 52°C to 61°C) in RT-PCR. (B) Gel electrophoresis of RT-qPCR products of reference genes. M: DNA marker.
(TIF)

S4 Fig Expression profiles of miR169, miR172, miR838, miR854, miR2673, miR5637, miR5658 and csa-miRn6-3p and validation of selected reference genes in cucumber leaf tissues infected with CGMMV. Error bars represent the mean of three technical replicates ± SD.
(TIF)

S1 File Sequences of candidate reference genes. (PDF)
