# Supplementary figures and images for: Identifying optimal reference genes for the normalization of microRNA expression in cucumber under viral stress
Source: PLoS One. 2018 Mar 15;13(3):e0194436. doi: 10.1371/journal.pone.0194436 (PMC5854380; doi:10.1371/journal.pone.0194436)

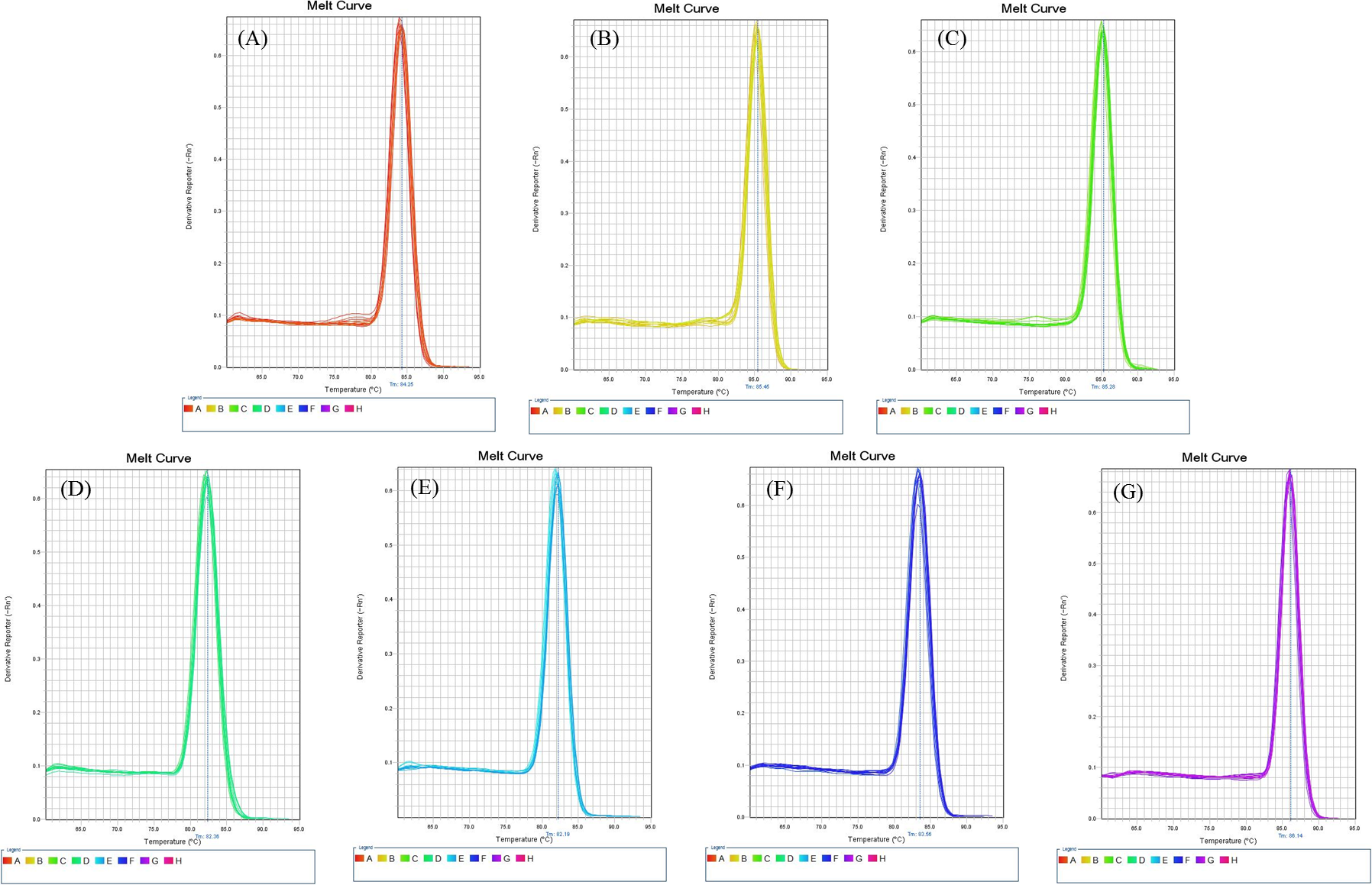

Supplement: S1 Fig — (A) to (G) Melting curves of Actin, Tubulin, EF-1α, 18S rRNA, Ubiquitin, GAPDH and Cyclophilin. (TIF) [file pone.0194436.s001.tif]

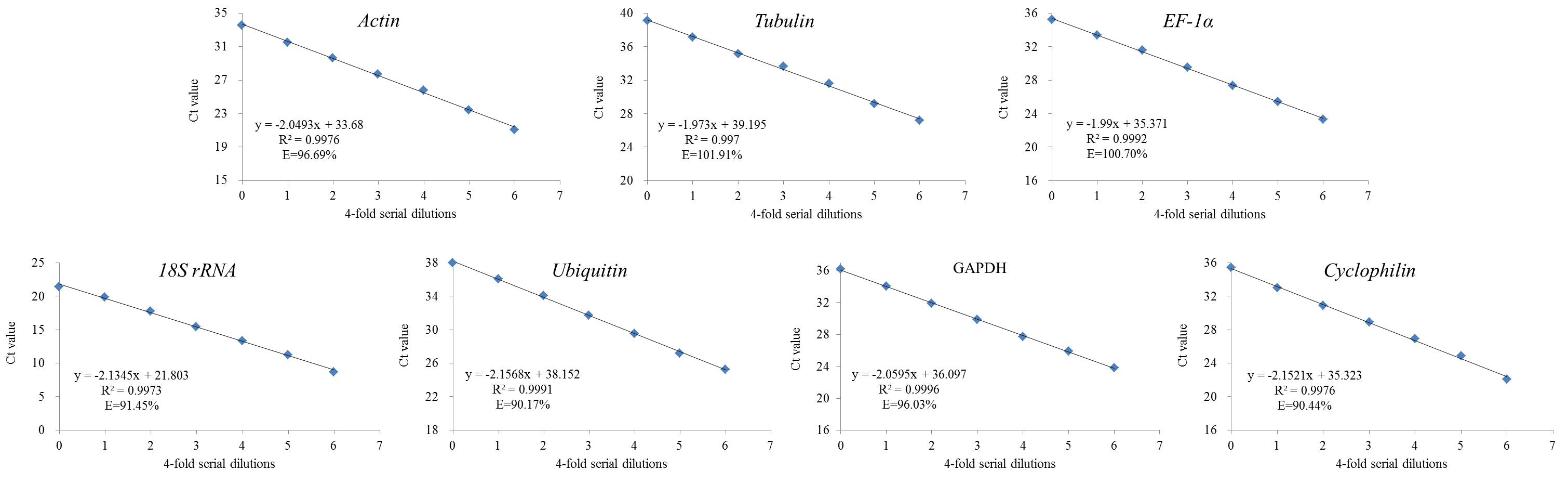

Supplement: S2 Fig — (TIF) [file pone.0194436.s002.tif]

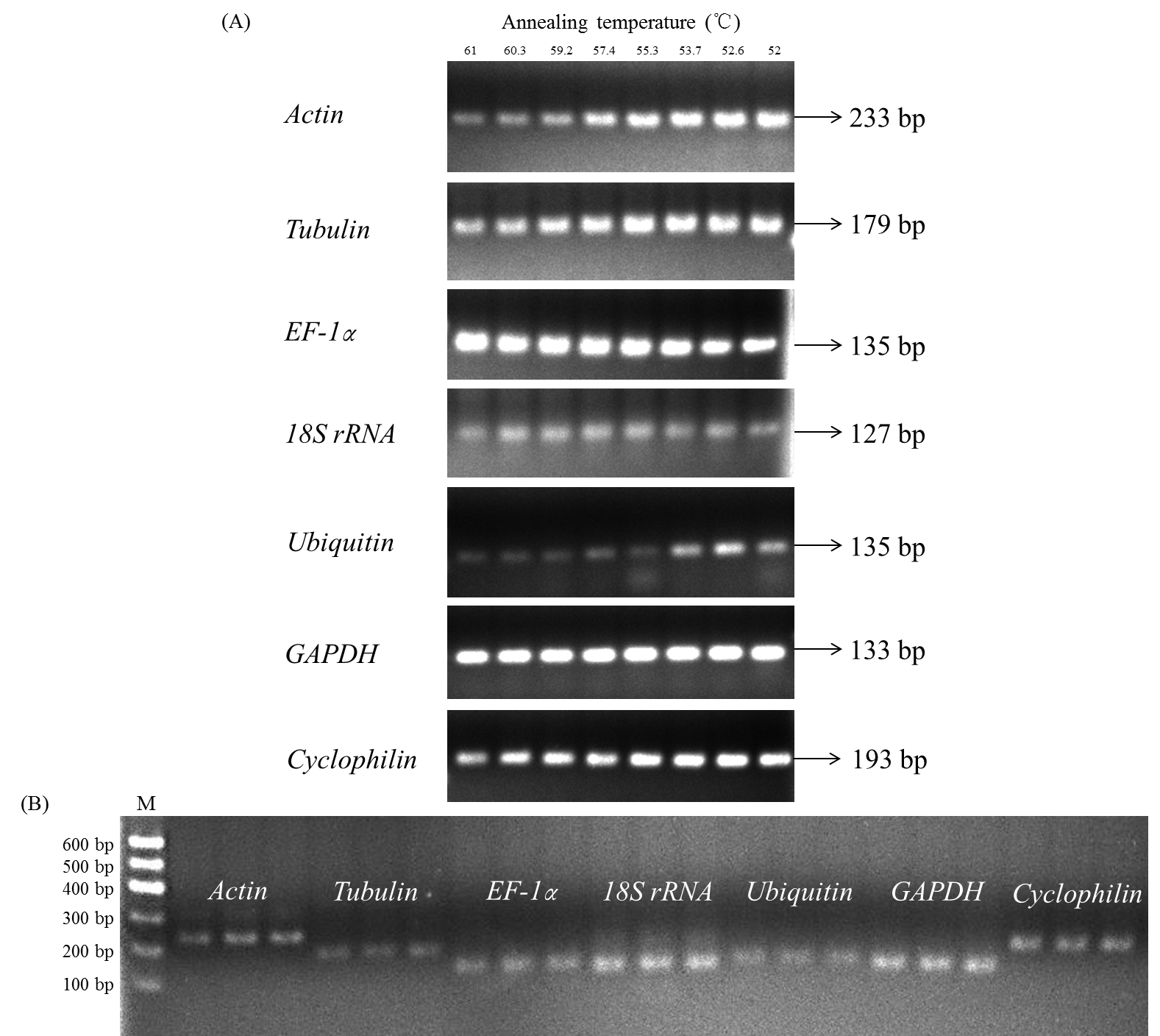

Supplement: S3 Fig — (A) Amplification of reference genes with gradient annealing temperatures (from 52°C to 61°C) in RT-PCR. (B) Gel electrophoresis of RT-qPCR products of reference genes. M: DNA marker. (TIF) [file pone.0194436.s003.tif]

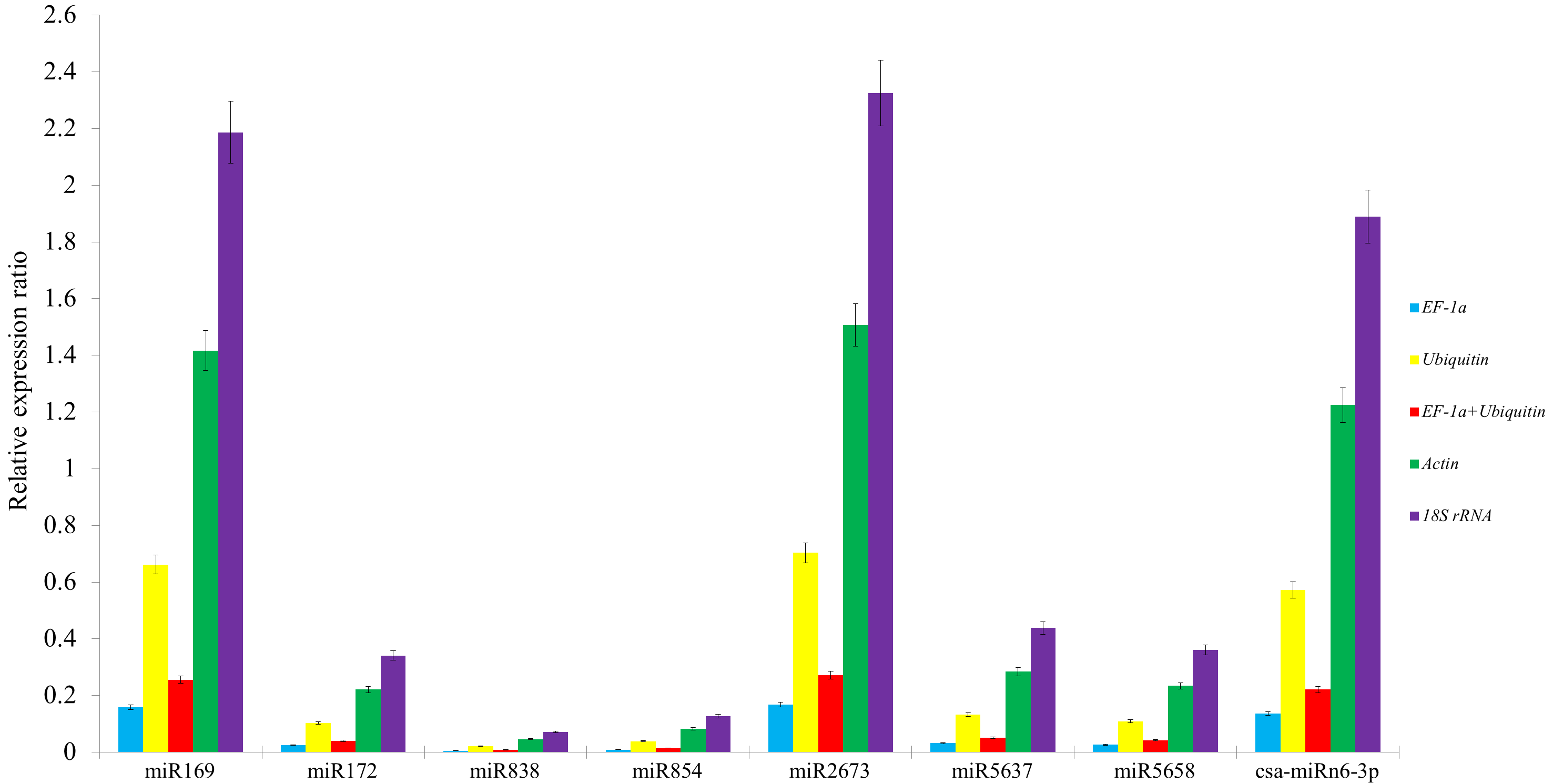

Supplement: S4 Fig — Error bars represent the mean of three technical replicates ± SD. (TIF) [file pone.0194436.s004.tif]
