# Supplementary material for: Identifying optimal reference genes for the normalization of microRNA expression in cucumber under viral stress
Source: PLoS One. 2018 Mar 15;13(3):e0194436. doi: 10.1371/journal.pone.0194436 (PMC5854380; doi:10.1371/journal.pone.0194436)
Supplement: S1 File — (PDF) [file pone.0194436.s005.pdf]

1.Actin (AB010922.1)
GAAAAGATGACGCAGATAATGTTTGAGACATTCAATGTGCCTGCTATGTATGTTGCCATCCAGGCCGTT
CTGTCCCTCTACGCTAGTGGACGTACCACTGGTATCGTGCTGGATTCTGGTGATGGTGTGAGTCACACT
GTTCCCATCTATGAGGGTTACGCCCTCCCTCATGCCATTCTCCGTTTGGACCTTGCTGGTCGTGACCTTA
CTGATGCTCTCATGAAAATTCTCACTGAAAGAGGTTACATGTTCACCACCACTGCCGAACGGGAAATTG
TCCGTGACATGAAAGAGAAGCTGGCATATGTTGCTCTTGACTACGAACAAGAACTCGAGACTGCAAAG
AGCAGTTCCTCTATTGAGAAGAACTATGAACTTCCTGATGGACAAGTCATCACCATCGGAGCTGAGAGA
TTCCGTTGCCCAGAAGTTCTATTCCAGCCATCTCTCATCGGTATGGAA

2.Tubulin (AJ715498.1)
TACCGCCAGCTCTTCCACCCTGAACAGCTGATCAGCGGCAAGGAAGATGCTGCCAATAATTTTGCACGT
GGCCACTACACCGTTGGAAAGGAAATTGTTGATCTCTGCTTGGACAGAATCCGCAAGCTTGCTGACAA
CTGCACTGGTCTTCAAGGATTCCTTGTTTTCAACGCTGTTGGTGGTGGTACCGGTTCTGGTCTCGGCTC
CCTCCTTTTGGAGCGTTTGTCTGTTGACTATGGAAAGAAATCCAAGCTTGGATTCACTGTTTACCCCTCT
CCACAAGTCTCAACTTCTGTCGTTGAGCCTTACAACAGTGTTCTCTCAACCCATTCTCTCTTGGAACAC
ACTGACGTTGCTGTGCTTCTTGACAACGAAGCCATTTATGATATCTGCAGGCGTTCCCTCGACATTGAG
CGACCTAACTACTCGAACCTCAACCGTCTTGTATCCCAGGTCATTTCATCTTTAACTGCCAGTTTGAGGT
TTGATGGTGCATTGAACGTGGATGTTAACGGGTTCCAGACCAACTTGGTCCCATATCCCAGAATCCAGT
TCATGCTCTCCTCATATGC

3. EF1α (EF446145.1)
GGAGGTATTGACAAGCGTGTGATTGAGAGATTCGAGAAGGAAGCCGCTGAGATGAACAAGAGGTCAT
TCAAGTATGCTTGGGTGCTCGACAAACTTAAGGCAGAGCGTGAACGTGGTATTACCATTGACATTGCCC
TGTGGAAGTTTGAGACCACCAAGTACTACTGCACAGTCATTGATGCTCCCGGACATCGTGACTTTATCA
AGAACATGATTACTGGAACCTCACAGGCTGACTGTGCTGTCCTCATTATTGACTCCACCACTGGTGGTT
TTGAGGCTGGTATTTCCAAGGATGGTCAGACCCGTGAGCACGCTCTTCTTGCTTTCACCCTTGGTGTCA
AGCAAATGATCTGCTGCTGCAACAAGATGGATGCCACCACACCCAAATACTCCAAGGCAAGGTACGAT
GAAATTGTGAAGGAAGTCTCATCATACCTCAAGAAGGTCGGATACAACCCAGACAAAATCCCCTTCGT
TCCCATCTCTGGTTTCGAGGGTGACAACATGATTGAGAGGTCCACCAACCTTGATTGGTACAAGGGAC
CAACTCTCC

4. 18S rRNA (AF206894.1)
TCATATGCTTGTCTCAAAGACTAAGCCATGCATGTGTAAGTATGAACTAATTCAGACTGTGAAACTGCG
AATGGCTCATTAAATCAGTTATAGTTTGTTTGATGGTATCTGCTACTCGGATAACCGTAGTAATTCTAGAG
CTAATACGTGCAACAAACCCCGACTTTCTGGAAGGGACGCATTTATTAGATAAAAGGTCGACACGGGC
TCTGCCCGTTGCTCTGATGATTCATGATAACTCGACGGATCGCACGGCCATCGTGCTGGCGACGCATCA
TTCAAATTTCTGCCCTATCAACTTTCGATGGTAGGATAGTGNCCTACTATGGTGGTGACGGGTGACGGA
GAATTAGGGTTCGATTCCGGAGAGGGAGCCTGAGAAACGGCTACCACATCCAAGGAAGGCAGCAGGC
GCGCAAATTACCCAATCCTGACACGGGGAGGTAGTGACAATAAATAACAATACCGGGCTCTTCGAGTCT
GGTAATTGGAATGAGTACAATCTAAATCCCTTAACGAGGATCAATTGGAGGGCAAGTCTGGTGCCAGCA
GCCGCGGTAATTCCAGCTCCAATAGCGTATATTTAAGTTGTTGCAGTTAAAAAGCTCGTAGTTGGACCTT
GGGTTGGGTCGATCGGTCCGCCTATGGTGAGCACCGGTCGGCTCGTCCCTTCTGCCGGCGATGCGCTCC
TGGCCTTAACTGGCCGGGTCGTGCCTCCGGCGCTGTTACTTTGAAGAAATTAGAGTGCTCAAAGCAAG
CCTACGCTCTGTATACATTAGCATGGGATAACGTCATAGGATTTCGATCCTATTTTGTTGGCCTTCGGGAT

CGGAGTAATGATTAACAGGGACAGTCGGGGGCATTCGTATTTCATAGTCAGAGGTGAAATTCTTGGATT
TATGAAAGACGAACAACTGCGAAAGCATTTGCCAAGGATGTTTTCATTAATCAAGAACGAAAGTTGGG
GGNTCGAAGACGATCAGATACCGTCCTAGTCTCAACCATAAACGATGCCGACCAGGGATTGGCGGATG
TTGCTTTAAGGACTCCGCCAGCACCTTATGAGAAATCAAAGTCTTTGGGTTCCGGGGGGAGTATGGTCG
CNAGGCTGAAACTTAAAGGAATTGACGGAAGGGCACCACCAGGAGTGGAGCCTGCGGCTTAATTTGA
CTCAACACGGGGAAACTTACCAGGTCCAGACATAGTAAGGATTGACAGACTGAGAGCTCTTTCTTGAT
TCTATGGGTGGTGGTGCATGGCCGTTCTTAGTTGGTGGAGCGATTTGTCTGGTTAATTCCGTTAACGAAC
GAGACCTCAGCCTGCTAACTAGCTATGCGGAGGTACCCCTCCGCGGCCAGCTTCTTAGAGGGACTATG
GCCGCTTAGGCCAAGGAAGTTTGAGGCAATAACAGGTCTGTGATGCCCTTAGATGTTCTGGGCCGCAC
GCGCGCTACACTGATGTATTCAACGAGTCTATAGCCTTGGCCGACAGGCCCGGGTAATCTTTGAAATTT
CATCGTGATGGGGATAGATCATTGCAATTGTTGGTCTTCAACGAGGAATTCCTAGTAAGCGCGAGTCAT
CAGCTCGCGTTGACTACGTCCCTGCCCTTTGTACACACCGCCCGTCGCTCCTACCGATTGAATGGTCCG
GTGAAGTGTTCGGATCGCGGCGACNTNGGCGGTTCGCTGCCNNCGANGTCNCGAGAAGTCCACTGAN
CCTTATCATT

5. Ubiquitin (AF104391.1)
GATTTCATCGCGATGAATTGAAGGAACGAACTTTCCCAAAGCACAAGCAAGAGACTTCTCGCTGATTC
CCAGACAGCGGTAGTGGAGGCAGTGTTTTATTGAAGTGTTGGTCCACTAGTTCTTGTTTCAAGTCAACT
ATGGCAATGTATATCCGAATCAAGCGCCATAAGACAACCTACTTTATCCAGTGTGATCCAATTGAGACAA
CTTTAAATATCAAGCAAAAATTAGAGTCCCTTATTGACCAACCAGTAGTTGACCAGCGCTTGATCCTAAT
GGGGAGTGGGGAAGTATTGGAGGATTCAAAGACACTGGCTGATCAGAAGGTTGAAAATGATGCAGTT
GTGGCTCTAACGTTGCGAAAAGATGATAATGACTTCGAGGAAATCAACATTGTCCATCCAGACGATTTC
TACCAATCACCGATTCTGGCGGTTGGTAAGGACTAAAAAAATTGCTTCYACTGTGGGTAGTATATGATGT
TCACTCATCAATTTACTGTTTAAACCACTTTTGTATCTGTCGAGTGGAAATGAAAATGATGAAAAAGTCT
ATGCATGATTATATATTGATTATTCCATGCTTTCTATTTGTTACCCTTTTAAGTGATAACCTCCAAGAGTTG
CATTAGTGATTGAACTCATCRATGTATRGTTGTTACAACTACTAAATATGAGTTGAGGGTTTTGAATAAA
AAAAAAAAAAAAAAAA

6. GAPDH (NM_001305758.1)
ATGGCTACGGCTACTCTGTCAGTAGCCAAACCATCTATTCAGGCTAATGGAAAGGGATTTGCAGAATTC
TCGGGCCTCCGCAACTCGTCGACCAGCCTTTCCTTTGCAAGGAGAACATCTGATGATTTCCTTTCCGTC
ATTGCCTTCCAAACCTCTGCAGTGGGAAGCAGTGGAGGATACAAGAAAGGGATTGTGGAAGCAAAGC
TTAAGGTAGCCATCAATGGGTTTGGAAGAATTGGCAGGAATTTCTTGAGGTGCTGGCATGGACGCAAG
GATTCCCCACTTGATGTCATTGCCATCAACGATACCGGAGGCGTCAAGCAAGCTTCTCACCTCCTCAAA
TACGATTCCACCCTCGGCATCTTTGAAGCTGATGTGAAACCTGCCGGAGATGAAGCCATTTCAGTCGAT
GGCAAGATCATCAAAGTGGTTTCCAGCCGCAATCCCCTCAACCTTCCCTGGAAGGACATGGAAATAGA
CTTGGTGATTGAAGGAACTGGAGTGTTTGTTGATAGAGAGGGTGCAGGGAAGCACATTGAGGCTGGA
GCTAAGAAGGTCCTAATTACAGCACCTGGGAAAGGTGACATTCCAACCTACGTTGTTGGGGTTAATGCT
GATGCATACAGCCATGACGAGTCCATCATCAGCAATGCTTCTTGCACTACCAACTGCCTAGCTCCTTTTG
TCAAGGTCCTTGACCAGAAATTTGGTATCATCAAGGGAACAATGACTACCACTCACTCCTACACTGGTG
ACCAGAGGCTACTCGATGCCAGCCACCGTGACCTCAGGAGGGCAAGAGCTGCTGCACTCAACATTGTT
CCTACATCCACAGGAGCAGCCAAAGCTGTTGCCTTGGTCCTCCCTTCTCTTAAAGGAAAGCTCAATGG
GATTGCACTTCGTGTGCCCACTCCAAATGCTTCTGTTGTCGACCTTGTTGTCCAGGTTTCTAAGAAGAC
ATTCGCTGAAGAGGTGAATGCTGCATTCCGGGAAAGTGCTGAGAAGGAGCTCAACGGTATCCTCTCTG

TTTGTGATGAGCCCCTTGTTTCGGTCGATTTTAGGTGCTCTGATGTCTCCTCAACTGTCGACTCTTCCTT
GACTATGGTTATGGGGGATGACTTGGTGAAGGTTATTGCTTGGTATGATAATGAGTGGGGTTACTCTCAA
CGGGTTGTTGATTTGGCTGACATTGTTGCCAACAACTGGAAATGA

7. Cyclophilin (AY942800.1)
GGCACGAGGAAATTTCGGAACTCACAGAAACAATCCAAAAATGCCAAACCCTAAAGTTTTCTTCGACA
TGACAATCGGCGGCACACCGGCCGGCCGGATCATCATGGAGCTCTACGCCGATGTCACTCCCCGCACC
GCCGAGAACTTCCGTGCACTCTGCACTGGTGAGAAGGGAGTCGGCAAAGGCGGCAAACCCCTTCATT
ACAAAGGATCCTCCTTCCACCGTGTGATCCCTAATTTCATGTGCCAGGGAGGTGATTTCACCGCCGGAA
ATGGTACAGGAGGTGAATCAATCTACGGAGCCAAGTTCGCCGATGAGAACTTCATCAAGAAGCACACC
GGCCCTGGTATTCTATCCATGGCGAATGCTGGACCTGGAACCAACGGATCTCAGTTCTTCATTTGCACC
GCTAAGACCGATTGGCTCGATGGTAAGCACGTTGTGTTCGGTCAAGTCGTTGAGGGTATGAATGTGGTA
AAAGACGTTGAGAAAGTGGGTTCGAGCTCTGGAAGGACCTCGAAACCGGTTGTCATTGCCGATTGTG
GCCAGCTCTCTTAGATTAACAGATGCTAGCTCCATCGCCGTTGTCTGCACCGGCGCAATCGTATCTAAGA
TCAATTTCCTATTTGCGTGTGTTGTTTTTAACTTCTGCGAATATGGGTCTGTGTGAGTCTCCTTTTTTAGG
CTATTATGGGTCATGGTTTATGCTACTTCATGAGTTTATATGCATCTGCCATGTTAAATGGCGGTTTCGCA
ATGGATTATTAATAATACATGGCTGTCTTTTAACTCAAAAAAAAAAAAAAAAAAAAA
